# Climate change advocacy and cancer clinical trial organisations

**DOI:** 10.1038/s44276-024-00074-6

**Published:** 2024-07-02

**Authors:** Nay My Oo, Catherine S. Weadick, Lucy Murphy, Seamus O’Reilly

**Affiliations:** 1 Department of Medical Oncology, Cork University Hospital, Wilton, Cork Ireland; 2 Cancer Trials Ireland, Royal College of Surgeons, St Stephens Green, Dublin, Ireland; 3 Cancer Research @UCC, College of Medicine and Health, University College Cork, Cork, Ireland

## Abstract

**Background:**

Climate change is a threat to human health; equally health care is a threat to climate change as it accounts for 4% of greenhouse gas emissions and 30% of the world’s electronically stored data. 350,000 international trials are registered on ClinicalTrials.gov with ~27·5 million tonnes of emissions (equivalent to half of annual Danish emissions).

**Methods:**

In September 2023 climate awareness among cancer clinical trial organisations was assessed via a web-based scoping exercise.

**Results:**

Seventy-five organisations were identified of whom 46 had search tools on their websites. Eight out of 46 clinical trial groups had at least one parameter of commitment to climate change, and 38 organisations had none. Of 46 websites, 5 had climate change position statements or policies, 4 had a committee or task force, 1 provided patient education resources for climate change via video link, 7 included green initiative advice and 8 had publications addressing climate change. Only 5 were listed as members of Climate Change Consortiums.

**Conclusions:**

Based on website assessment climate advocacy among cancer clinical trial organisations is low, and efforts to encourage climate engagement are needed.

## Background

A consensus statement from the Regional Action on Climate Change Symposium warned in 2022 that “the Earth’s climatic, ecological and human systems are converging towards a crisis that threatens to engulf global civilization within the lifetimes of children now living” [1]. Climate change poses significant risks for cancer incidence, care delivery and outcomes [2]. It also poses significant risk to cancer clinical trials by disrupting trial integrity through extreme weather events, wildfires and a spread of vector borne diseases [3], and by compromising funding resources.

Equally health care has a substantial environmental impact (and therefore cancer impact) as it’s very energy-intensive, and consequently is the fifth largest greenhouse gas emitter on the planet, accounting for over 4% of worldwide emissions [4]. Globally this equates to the emissions of the continent of Africa which has 1.5 billion people among 54 countries. Within healthcare, clinical trials have a carbon footprint equivalent to 50% of the annual footprint of Denmark, while the footprint of scientific discovery has the same annual footprint as Venezuela [5]. Integration of climate smart strategies in the clinical trial arena could mitigate against climate change, and subsequently create and embed a climate responsible ecosystem within clinical trials. Many of these organisations are partnered with each other and with academic institutions, and collaborate with industrial partners, all of which facilitates interdisciplinary innovation and positive disruptive climate-smart change.

## Methods

**Fig. 1:**
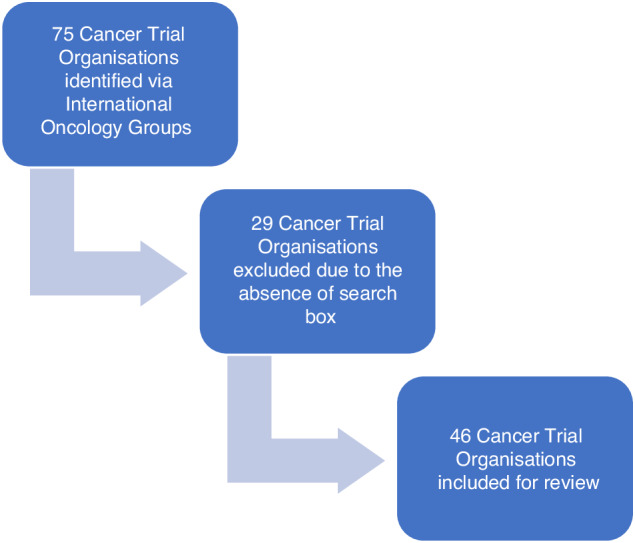
Selection Criteria for Cancer Trial Organisations.

We assessed the current activities of clinical trial organisations to reduce their carbon footprint and address the healthcare risks of climate change. We conducted a review of public facing websites of clinical cancer trial organisations in Europe, North America, Latin America, Asia and the Middle East between 1st and 9th of September 2023. Climate change parameters were assessed using a template devised by Bush et al. 2022 [3], in their assessment of the public facing websites of medical organisations in the United States. The metrics they chose were based on characteristics deemed to be most relevant for assessing an organisation’s commitment to climate change. Keyword searches were conducted by entering the terms “green initiative”, “climate change”, “global warming”, “carbon footprint”, “sustainable energy” and “carbon emission”. Since the search box was a key tool for identifying relevant content on a website, organisations without a functional search box were excluded (Fig. 1).

Each climate change parameter was assigned a point: (a) the inclusion of climate change into position statement or policy, (b) the establishment or committee or task force to promote sustainable healthcare, (c) membership in the consortium on sustainable healthcare or climate change, (d) educating patients regarding climate change, (e) recommendations on green initiative and how to mitigate climate change and (f) publications related to climate change on the websites. To award maximum points for any contribution or commitment made by cancer trial organisations, we used a non-weighted scoring system. The maximum score that could be achieved if all parameters were met was 6 points.

## Results

We identified a total of 75 cancer trial organisations based on their affiliation with International Cancer Trial Groups such as Breast International Group (BIG), EORTC (European Organisation for Research and Treatment of Cancer) and SWOG (Southwest Oncology Group). After excluding the websites that lacked a functional search box, only 46 organisations were available for review.

We then searched each of the 46 organisations’ websites for 6 metrics regarding the climate change (position statements or policies, committees, patient education, recommendations on green initiatives, publications and partnership with climate change organisations or consortiums) for the total highest possible 276 points (46 × 6). Two climate change organisations were found to have linked with a few cancer trial groups: Sustainable Healthcare Coalition and Helmholtz Association of German Research Centres. Some organisations’ websites include a link to educational videos on climate change and are considered as patient education materials to assign one metric point.

The scores of Cancer Trial Organisations on metrics assigned to each component of climate change commitment are presented in Table 1. With maximum obtainable score of 6 points (100%), the total scores of each cancer trial organisation ranged from 0 to 6 (0–100%). Eight organisations were found to have at least one metric point for climate commitment while 38 had no evidence of such content related to climate change. Four organisations scored greater than 80%, Cancer Research UK, Medical Research Council, National Institute for Health and Care Research, and The German Cancer Research Center (DKFZ).

**Table 1:** Evaluation of Website of Cancer Clinical Trials Organisations.

Cancer Trial Organisation	Position Statement/ Policy	Committee	Patient Education	Publication	Practice Recommended Action	Member of a Consortium	Total Score (percentage)
United Kingdom							
Cancer Research UK	1	1	0	1	1	1	5 (83%)
Cancer Research Wales	0	0	0	0	0	0	0
ECMC (experimental cancer medicine centres)	0	0	0	0	0	0	0
ICR-CTSU (Institute of Cancer Research – Clinical Trials & Statistics Unit)	1	0	0	1	1	1	4 (67%)
MRC (Medical Research Council)	1	1	0	1	1	1	5 (83%)
Thames Valley Cancer Network	0	0	0	0	0	0	0
NIHR (National Institute for Health and Care Research)	1	1	0	1	1	1	5 (83%)
EUROPE							
AIO (Arbeitsgemeinschaft Internistische Onkologie)	0	0	0	0	0	0	0
AIRC (Associazione Italiana per la Ricerca sul Cancro)	0	0	0	0	0	0	0
ABCSG (Austrian Breast and Colorectal Cancer Study Group)	0	0	0	0	0	0	0
BOOG (Borstkanker Onderzoek Groep)	0	0	0	0	0	0	0
CEEOG (Central and East European Oncology Group)	0	0	0	0	0	0	0
Cancer Trials Ireland	1	0	0	0	1	0	2 (33%)
DBCG (Danish Breast Cancer Group)	0	0	0	0	0	0	0
DKFZ (The German Cancer Research Centre)	1	1	0	1	1	1	5 (83%)
EBMT (European Bone Marrow Transplantation)	0	0	0	0	0	0	0
EORTC (European Organisation for Research and Treatment of Cancer)	0	0	0	0	0	0	0
EORTC BCG (EORTC Breast Cancer Group)	0	0	0	0	0	0	0
ETOP (European Thoracic Oncology Platform)	0	0	0	0	0	0	0
GBG (German Breast Group)	0	0	0	0	0	0	0
GCIG (Gynaecologic Cancer Inter Group)	0	0	0	0	0	0	0
GEICAM (Spanish Breast Cancer Group)	0	0	0	0	0	0	0
HeCOG (Hellenic Cooperative Oncology Group)	0	0	0	0	0	0	0
IBCSG (International Breast Cancer study group)	0	0	0	0	0	0	0
IEO (Instituto Europeo di Oncologia)	0	0	0	0	0	0	0
IJB-CTSU (Institut Jules Bordet Clinical Trials Support Unit)	0	0	0	0	0	0	0
MICHELANGELO (Fondazione Michelangelo)	0	0	0	0	0	0	0
NBCG (Norwegian Breast Cancer Group)	0	0	0	0	0	0	0
NUC (Nordic Cancer Union)	0	0	0	0	0	0	0
SAKK (Swiss Group for Clinical Cancer Research)	0	0	0	0	0	0	0
SOLTI Breast Cancer Research	0	0	0	0	0	0	0
UCBG (Unicancer Group, Unicancer Breast Cancer Group)	0	0	0	0	0	0	0
WSG (West German Study Group)	0	0	0	0	0	0	0
ASIA							
CTRG (Cancer Therapeutic Research Group)	0	0	0	0	0	0	0
NCG (National Cancer Grid)	0	0	0	0	0	0	0
SKMCH & RC (Shaukat Khanum Memorial Cancer Hospital & research Centre)	0	0	0	0	0	0	0
AUTRALIA							
Australasian Gastrointestinal Trial Group (AGITG)	0	0	0	0	0	0	0
ANZGOG (Australian New Zealand Gynaecological Oncology Group)	0	0	0	0	0	0	0
BCT-ANZ (Breast Cancer Trial Australia and New Zealand)	0	0	0	0	0	0	0
TROG (Trans-Tasman Radiation Oncology Group)	0	0	0	0	0	0	0
LATIN AMERICA							
GECO PERU (Grupo de Estudios Clinicos Oncologicos Peruano)	0	0	0	0	0	0	0
GOCUR (Grupo Oncológico Cooperativo Uruguayo)	0	0	0	0	0	0	0
NORTH AMERICA							
AACR (American Association for Cancer Research)	0	0	0	1	0	0	1 (17%)
CCTG (Canadian Cancer Trials Group)	0	0	0	0	0	0	0
NCI (National Cancer Institute)	0	0	1	1	1	0	3 (50%)
SWOG (South West Oncology Group)	0	0	0	0	0	0	0

Position statements or policies related to climate change awareness were found in 5 organisations. Committees or task forces to tackle climate change were found in 4 websites. One cancer trial organisation provided patient education resources. Publications related to climate change were found in the websites of 8 organisations. Five organisations were listed as members of Climate Change Committed associations: Sustainable Healthcare Coalition and Helmholtz Association of German Research Centre. Most Cancer Trial Organisations addressed the impact of climate change on cancer care and research and reducing carbon emissions to achieve sustainable health care.

## Discussion

Our results are concordant with similar studies of medical organisations in the United States reviewed in March 2022 [3], in which 50 of 111 organisations had at least one metric relevant to climate change, 22 websites had position statements or policies, 12 had task forces and 9 provided patient education. Fifteen were listed as member societies of the Medical Consortium on Climate Change. In a similar, predominantly web based, assessment of 64 National Cancer Institute cancer centers, 2 centers had independent sustainability plans and 11 centers reported on their sustainability efforts [2].

Cancer clinical trial organisations are critically placed to take a leading role in mitigating the climate change impact of health care. They are adept in protocol development and in the dissemination of patient appropriate information. They have demonstrated adaptability during the COVID-19 pandemic [6, 7], and with the successful integration of patient participation and involvement in routine trial conduct [8]. They have prioritized the development of less resource intensive (and consequently less climate toxic) treatment schedules [9–12], and their collaborative nature offer the potential for less redundancy in cancer discovery [13, 14]. Their focus on translating laboratory-based discovery into clinical care integrates them with a community where climate smart initiatives such as “My Green Lab” are already successfully embedded [15]. The nature of their funding models would allow successful integration of sustainability policies analogous to those used to promote gender equality in research [16, 17].

In the present study, four organizations had significant engagement in climate change. Three of these were from the United Kingdom where a recent workshop forum, jointly hosted by the Academy of Medical Sciences, the Medical Research Council and the National Institute for Health and Care Research, has highlighted four key challenges to enabling greener biomedical research. These include prioritising sustainability, generating and disseminating evidence on environmentally sustainable research practices, accelerating the introduction of environmentally sustainable practice in research and promoting and informing behavioural change [18]. In the clinical trials arena, there are multiple actionable areas including reducing research waste, encouraging our healthcare systems to reduce their carbon footprint, quantifying the carbon footprint of our clinical trials, green clinical trial initiatives, integrating sustainability practises into grant applications and integrating sustainability symposiums into medical conferences [18, 19]. Such developments are important. A multinational survey of 4654 healthcare professionals was conducted to assess their views on climate change as a human health issue, showed that awareness was high, but barriers existed to their engaging in advocacy and education on this issue [20]. Over 70% of respondents reported that policy statements by professional organisations and guidance documents on workplace sustainability would be helpful to them.

During the web search conducted for this study, the available search boxes were utilised to explore the activities of cancer trial organisations in relation to climate change. However, it is important to note that some organisations’ activities may not have been detected if their websites did not include a search box. Although extensive efforts were made to search for as many cancer trial organisations as possible, we acknowledge that not every organisation could be thoroughly reviewed due to the lack of a specific domain. Therefore, it is possible that certain organisations’ activities related to climate change were not captured in this review. An additional limitation is that we employed a non-weighted scoring system unlike that employed by Bush et al. [3]. This could conceal asymmetries in the level of commitment by the organisations surveyed and their engagement in this area in that in the method used policy statements and publications and equal weighting.

Our survey demonstrates a high level of climate-smart engagement in only 4 of 46 assessable cancer clinical trial organisations which is concordant with results reported from surveys of medical organisations. This website survey has prompted a survey of climate change impacts, sustainability engagement, barriers to and facilitators of engagement in sustainability initiatives among members of the Breast International Group [21]. The aim of the survey is to provide an evidence base for developing a sustainability initiative in this area. The results of the present website survey contrasts with sustainability engagement by the scientific community [22, 23], who have provided exemplars for engagement [15]. This community has significant interaction with the clinical trial community offering the potential to spiral this engagement from bench to bedside. The results of the present survey represent a window of opportunity to reduce the carbon footprint of discovery and embed climate awareness in the clinical trials community. The recent forum workshop provides a template for this engagement [18]. The occurrence of extreme weather events [24] in many of the countries where the organisations reviewed in this study are based highlights the urgent need for such engagement.

## Data Availability

Data sharing not applicable to this article as no datasets were generated or analysed during the current study.
